# Cyanide Poisoning

**DOI:** 10.21980/J80W76

**Published:** 2022-07-15

**Authors:** Ghadeer Doman, Jihad Aoun, Joshua Truscinski, Mariah Truscinski, Shaza Aouthmany

**Affiliations:** * King Abdulaziz University, College of Medicine, Department of Emergency Medicine, Jeddah, Saudi Arabia; ^ University of Toledo College of Medicine, Department of Emergency Medicine, Toledo, OH

## Abstract

**Audience:**

The goal of this simulation is to educate emergency medicine students, residents, attending physicians, and mid-level practitioners to recognize, diagnose, and manage acute cyanide toxicity.

**Introduction:**

Cyanide has an almond scent and is a naturally occurring compound. It is present within many different types of plants and fruits including apricots, apples, peaches, lima beans, and cassava plants but is harmless.^1^ The trace amounts of cyanide found within organic materials is of little concern because its high reactivity causes it to be metabolized rapidly and create other compounds. However, modern synthetic materials such as plastics, papers, textiles, and machinery can release a much greater concentration of hydrogen cyanide when exposed to high temperatures.^1^ As the use of contemporary nitrogen-containing synthetic polymers has expanded, the possibility of cyanide toxicity has become increasingly common and severe. Hydrogen cyanide is especially dangerous to humans because the gaseous form reacts quickly upon inhalation.^2^

When cyanide enters the body via inhalation, it blocks the cells from utilizing oxygen by binding to the cytochrome oxidase in the mitochondria.^2^ The inability of the cell to use oxygen forces cells from aerobic metabolism into anaerobic metabolism. Anaerobic metabolism results in the production of lactic acid, which causes metabolic acidosis.^3^ The human body cannot sustain itself with the lack of oxygen and anaerobic metabolism for a prolonged period of time. Ultimately, the body will suffer cardiorespiratory arrest.^1^

Symptoms of cyanide toxicity include headache, nausea, shortness of breath, and altered mental status.^1^ These are similar to those of carbon monoxide and carbon dioxide inhalation. However, symptoms of cyanide toxicity cannot be treated with supplemental oxygen as carbon monoxide and carbon dioxide are. Cyanide toxicity must be treated with an antidote - sodium thiosulfate, sodium nitrite, and hydroxocobalamin.^4^ Each of the antidotes works by binding with the highly reactive cyanide, neutralizing the compound, and converting it into a water-soluble product that will be cleared through renal excretion.^4^

Fire victims often present to the emergency department critically ill. They will likely have obvious external thermal burns and traumatic injuries; however, it is important for emergency personnel to recognize the respiratory distress and metabolic derangements that are most likely occurring due to toxic gas inhalation. People who are trapped within a burning structure are exposed to carbon monoxide, carbon dioxide, and cyanide from the combustion of contents within the building. These toxic gasses will cause severe tissue hypoxia without significant vital sign changes.^5^ The respiratory distress and metabolic compromise will be acutely more fatal than the obvious external injuries and burns. The challenge in treating these patients is for the healthcare team to know the differential diagnoses, prioritize airway, breathing and circulation, and to empirically treat the patient as if they have a confirmed exposure.

It is estimated that 35% of all fire victims have toxic levels of cyanide upon arrival to the emergency room.^2^ Acute cyanide toxicity can become fatal within minutes; however, a prompt diagnosis and treatment can be lifesaving. Unfortunately, due to the limited amount of time the human body can sustain anaerobic metabolism and tissue hypoxia, blood test results are not available in time to be clinically applicable.^2^ Rather, the emergency room personnel must begin treatment immediately upon recognizing that toxic smoke inhalation may have occurred.

We understand the importance of knowing how to treat fire victims. Therefore, the goal of this simulation case is to expose the emergency providers to cyanide poisoning and educate emergency providers about the critical steps of how to approach, diagnose, and treat cyanide toxicity.

**Educational Objectives:**

After the completion of this simulation, participants will have learned how to: 1) identify clues of smoke inhalation based on a physical examination; 2) identify smoke inhalation-induced airway compromise and perform definitive management; 3) create a differential diagnosis for victims of fire cyanide poisoning, carbon monoxide, and carbon dioxide; 4) appropriately treat cyanide poisoning; 5) demonstrate the importance of preemptively treating for cyanide poisoning; 6) perform an initial physical examination and identify physical marks suggesting the patient is a fire and smoke inhalation victim; and 7) familiarize themselves with the Cyanokit and treatment with hydroxocobalamin.

**Educational Methods:**

This is a high-fidelity simulation case in which participants work through a case of a patient who has been exposed to fire. The participants will be able to work hands-on to evaluate, diagnose, and treat cyanide poisoning in an emergency event. Afterwards, there will be a small group discussion and debriefing of the case in order to review patient care skills, interpersonal and communication skills, medical knowledge, and system-based practice.

**Research Methods:**

The participants were instructed to complete a survey before and after the simulation case. A quality Likert Scale was used to assess the participants’ comfort level of diagnosing, treating, and managing a patient with toxic smoke inhalation. A score of 1 represented a negative experience and 5 represented a very positive experience. The surveys were then reviewed by the research team to determine if the simulation case improved the participants’ comfort level. The survey answers were compared collectively, as well as individually, and were analyzed between the pre-simulation and post-simulation results.

**Results:**

Our simulation involved 25 participants: 20 participants were emergency medicine resident physicians and 5 were 4th-year medical students. In the pre-simulation survey, participants reported a mean of 2.7 out of 5 when asked to rate their confidence in their ability to treat a smoke inhalation victim. The post-simulation survey showed a significant increase to a mean of 3.5 out of 5. Participants were also asked to evaluate the usefulness of the simulation: 15 participants rated the case as a 5, which represented “very useful,” and the other 10 participants rated the case as a 4, which represented “useful.” The mean value when asked to assess the simulation case’s usefulness and applicability in emergency medicine was 4.6 out of 5.

**Discussion:**

This simulation allows providers to focus on victims of fire. Fire victims are often critically ill and require time sensitive treatment. This simulation gives providers a chance to review their knowledge and prepare them for real life cases. Based on the survey results, the simulation improved awareness and understanding of the symptoms of acute cyanide toxicity and improved the participant’s ability to recognize, diagnose, and treat cyanide poisoning.

**Topics:**

Cyanide toxicity, carbon monoxide toxicity, cyanide antidote, fire victim, intubation, airway intervention, oxygen treatment, history taking, lab testing ordering, symptom identification, interpretation of lab results, emergency medicine simulation.

## USER GUIDE

**Table t1-jetem-7-3-s1:** 

**List of Resources:**	
Abstract	1
User Guide	4
Instructor Materials	6
Operator Materials	16
Debriefing and Evaluation Pearls	19
Simulation Assessment	21


[Table t1-jetem-7-3-s1]

**Learner Audience:**

Medical students, resident physicians

**Time Required for Implementation:**

**Instructor Preparation:** 20–30 minutes

**Time for case:** 15–20 minutes

**Time for debriefing:** 10 minutes

**Recommended Number of Learners per Instructor:** 2–5

**Topics:**

Cyanide toxicity, carbon monoxide toxicity, cyanide antidote, fire victim, intubation, airway intervention, oxygen treatment, history taking, lab testing ordering, symptom identification, interpretation of lab results, emergency medicine simulation.

**Objectives:**

At the conclusion of this simulation, participants will have learned to:

1. Identify clues of smoke inhalation based on a physical examination
2. Identify smoke inhalation-induced airway compromise and perform definitive management
3. Create a differential diagnosis for victims of fire - cyanide poisoning, carbon monoxide and carbon dioxide
4. Appropriately treat cyanide poisoning
5. Demonstrate the importance of preemptively treating for cyanide poisoning
6. Perform an initial physical examination and identify physical marks suggesting the patient is a fire and smoke inhalation victim.
7. Familiarize themselves with the Cyanokit and treatment with hydroxocobalamin

### Linked objectives and methods

This is a high-fidelity simulation case in which participants can work through a case of a patient who has been exposed to fire. The participants will be able to work hands-on to evaluate, diagnose, and treat cyanide poisoning in an emergency event. Learners will take a history and perform a physical exam to identify clues for diagnosis (objective 1 and 6). Appropriate labs and imaging should be ordered at this time. When learners identify smoke inhalation injury, definitive management should be performed (objective 2) and treatment for cyanide and/or carbon monoxide poisoning administered immediately (objective 3 and 4). The simulation will allow participants to review cyanide poisoning treatment and be reminded of the importance of treating cyanide poisoning preemptively since it is highly associated with mortality (Objective 5 and 7).

### Recommended pre-reading for instructor

- Breen P, Isserles S, Westley J, Roizen M, Taitelman U. Combined carbon monoxide and cyanide poisoning. *Anesth Analg*.1995;80(4):671–677. doi: 10.1097/00000539-199504000-00004
- Graham J, Traylor J. Cyanide Toxicity. [Updated 2020 Jul 2]. In: StatPearls [Internet]. Treasure Island (FL): StatPearls Publishing; 2020 Jan-. Available from: https://www.ncbi.nlm.nih.gov/books/NBK507796/

### Results and tips for successful implementation

This case will allow participants to treat a smoke-inhalation victim including airway management and preemptive treatment based on history and physical. By following the simulation events table (see below), participants will understand the importance of quick decision making to avoid the patient going into cardiac arrest.

The simulation was performed during weekly didactics for emergency medicine residents and medical students. The residents and medical students were split into groups of 4 or 5 and were presented the case by two physicians. The participants were graded on their ability to intubate the patient, create a differential diagnosis for a smoke inhalation victim, and successfully treat for cyanide poisoning. Participants also received emailed surveys before and after the simulation where they were able to offer feedback regarding the case design/implementation. The emailed surveys had two sections with the first section designed using a Likert scale to assess how useful and applicable the case was while the second section was a free response to allow participants to voice any changes or issues with the simulation that they would like corrected. Of the participants, 60% rated the case as “very useful” (the highest rating possible), while the remaining 40% rated the case as “useful” (the second highest rating possible). Along with email surveys, the debriefing session was held directly following the simulation where the physician presenters spoke with participants, answered questions, and addressed any mistakes made by the participants. During the debriefing session, participants were also asked if they would make any alterations to the case. Overall, participants found the simulation helpful and applicable, and through the information collected during the debriefing session and the email survey, participants did not recommend any significant changes to the design or implementation of the simulation.

## Supplementary Information





















## Figures and Tables

**Table t2-jetem-7-3-s1:** 

#1	Complete Blood Count (CBC)
#2	Comprehensive Metabolic Panel (CMP)
#3	Phosphorus and Magnesium
#4	Troponin
#5	Arterial Blood Gas (ABG) Test with Co-ox
#6	Lactic Acid Test
#7	Urinalysis
#8	Urine tox screen
#9	Electrocardiogram (ECG)
#10	Chest Radiograph (CXR)
#11	Computed Tomography (CT) - Chest, Abdomen & Pelvis
#12	Ankle Radiograph (X-ray)
#13	Cyanide level
#14	Carbon monoxide level
#15	Cervical Spine Collar

**Table t3-jetem-7-3-s1:** 

**Vitals:**	Heart Rate (HR) 132	Blood Pressure (BP) 107/75 right arm
	Respiratory Rate (RR)	24 Temperature (T) 37.5°F
	Oxygen Saturation (O_2_ Sat) 98% on 6L NC	

**Table t4-jetem-7-3-s1:** 

*Complete blood count (CBC)*	
White blood count (WBC)	7.9 **×** 1000/mm^3^
Red Blood Count (RBC)	5.0 **×** 10^6^/mm^3^
Hemoglobin (Hgb)	16.1 g/dL
Hematocrit (HCT)	45.6%
Mean Corpuscular Volume (MCV)	87
Platelet (Plt)	180 **×** 1000/mm^3^

**Table t5-jetem-7-3-s1:** 

*Comprehensive metabolic panel (CMP)*	
Sodium	140 mEq/L
Potassium	4.2 mEq/L
Chloride	103 mEq/L
Blood Urea Nitrogen (BUN)	17 mg/dL
Creatinine (Cr)	1.05 mg/dL
Glucose	88 mg/dL
Calcium	9.9 mg/dL
Total bilirubin	0.6 mg/dL
Alkaline phosphatase	71 units/L
Aspartate aminotransferase (AST)	16 units/L
Alanine aminotransferase (ALT)	30 units/L
Total Protein	7.1 g/dL
Albumin	4.5 g/dL
Phosphorous	3.2 mg/dL
Magnesium	1.6 mEq/L

**Table t6-jetem-7-3-s1:** 

*Arterial Blood Gas (ABG)*	
pH	7.10
pCO2	20 mEq/L
pO2	92 mEq/L
Bicarbonate (HCO_3_)	14 mEq/L
Carboxyhemoglobin	8%
*Lactic Acid*	*15.1 mmol/L*
*Cyanide Level*	*2.0 mcg/mL*

**Table t7-jetem-7-3-s1:** 

*Urine Toxicology Screen:*	
Amphetamines	*Negative*
Barbiturates	*Negative*
Benzodiazepines	*Negative*
Methadone	*Negative*
Cocaine	*Negative*
Opiates	*Negative*
Phencyclidine (PCP)	*Negative*
Tetrahydrocannabinol (THC)	*Negative*

**Figure f1-jetem-7-3-s1:**
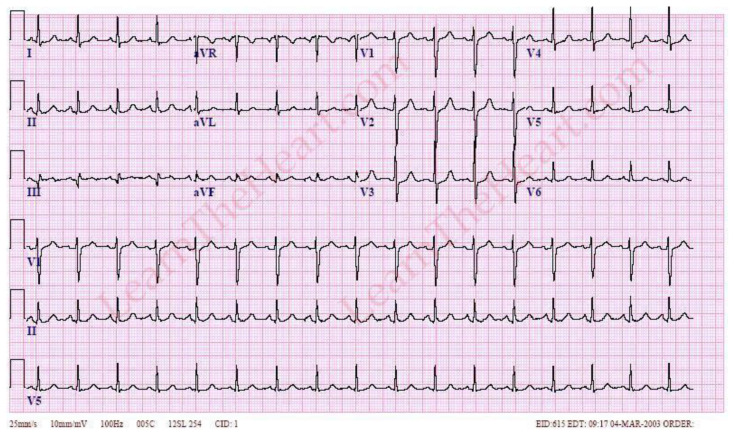


**Figure f2-jetem-7-3-s1:**
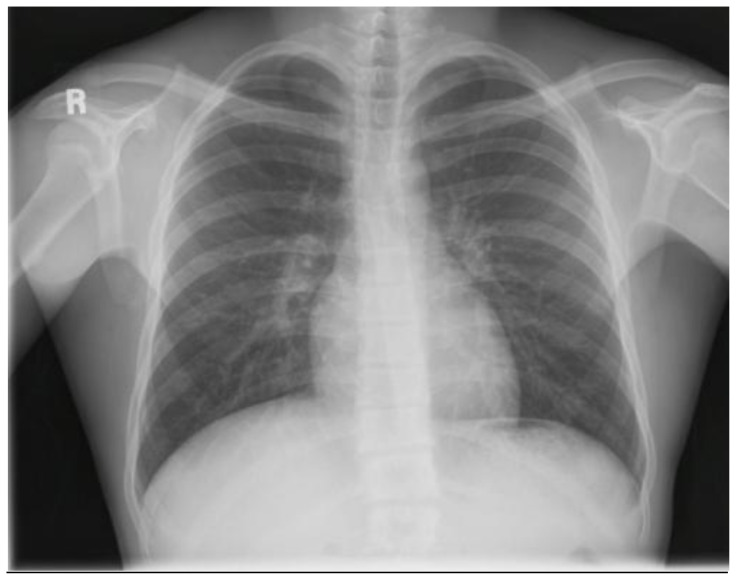


**Figure f3-jetem-7-3-s1:**
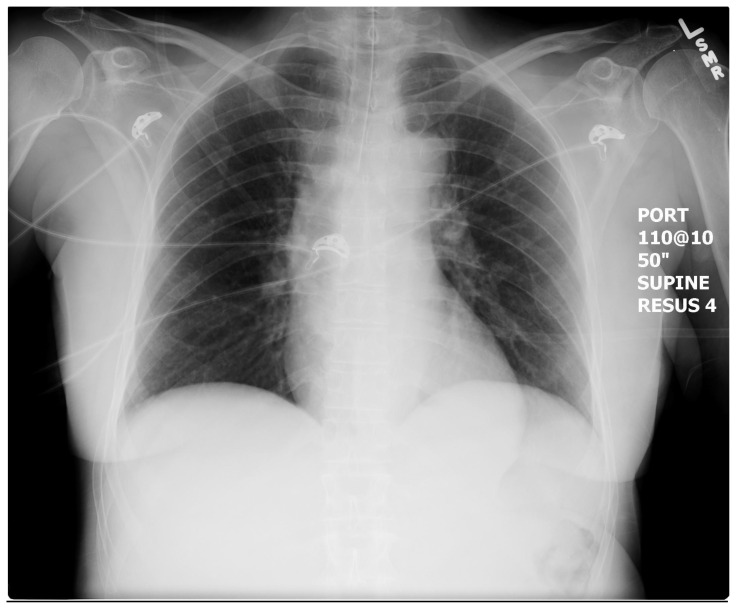


**Table t8-jetem-7-3-s1:** SIMULATION EVENTS TABLE:

Minute (state)	Participant action/trigger	Patient status (simulator response) & operator prompts	Monitor display (vital signs)
**State 1: Arrival to ED**	- Patient arrived to the ED via EMS - Participant should request nurse to place patient on monitor, have IV placed and blood work drawn - Respiratory therapist raises concern about breathing (tachypnea, stridor) and requests intervention *** if patient is intubated, go to State 3 *** if patient is placed on noninvasive oxygen, go to State 2	- Airway intact - patient able to state his name but stridor present at rest - Breathing - Patient has bilateral wheezing on auscultation - Circulation - 2+ pulses x4 extremities; tachycardia - patient should be started on IV fluids - Patient’s skin appears very red **- Transition:** Once airway is addressed, go to next state; if non-invasive oxygen is chosen, go to State 2; if intubation, go to State 3	T: 37.5° C HR: 132 BP: 107/75 RR: 24 SpO2: 98% on 6L ECG (rhythm): Sinus tachycardia
**State 2:**	- Participants should identify worsening respiratory distress (increased RR, decreasing SpO2) and worsening vitals (worsening tachycardia and hypotension) - Participants should be able to recognize that carbon monoxide toxicity is likely not causing symptoms based on 98% oxygen saturation. -Participants should create a differential diagnosis	- Airway– louder stridor, increasing HR - Mentation worsening - patient is becoming confused - **Transition:** Once patient is intubated, go to Step 3; if patient is not intubated, patient will cardiorespiratory arrest	T: 37.5° C HR: 150 BP: 98/71 RR: 28 SpO2: 96% ECG (rhythm): Sinus tachycardia
**State 3: Patient intubated**	- Post intubation vitals should be reviewed - Participants should complete disability and exposure - Differential diagnosis should be discussed - Post intubation X-ray should be ordered	- Skin is still red - Blood pressure will mildly decrease due to post intubation - Exposure/Disability: singed facial hair, soot in nose, red skin but no extremity burns, obvious closed right ankle deformity **- Transition:** Give cyanide antidote; if any other test/procedure/consult is requested, patient will cardiac arrest	T: 37.5° C HR: 120 BP: 90/62 RR: 20 SpO2: 97% ECG (rhythm): Sinus tachycardia
**State 4: Stabilization**	- Completion of secondary assessment - right ankle X-ray, EKG, lab results given - Ankle stabilized - Critical care contacted for admission	- Repeat Chest X-ray will show proper tube placement, but no infiltrate. - Lab results are finalized **Case Closed**	T: 37.5° C HR: 101 BP: 110/81 RR: 20 SpO2: 99% ECG (rhythm): Sinus tachycardia

**Table t9-jetem-7-3-s1:** 

**Critical Actions:** Obtain full history from the patient/EMS Obtain Vital Signs, recognize abnormalities and address supplemental oxygen fluids for tachycardia Identify respiratory distress and increase oxygen support Intubation will be required Address the possibility of cyanide poisoning Consult Poison Control Center and treat with antidote Secondary Assessment - Order proper EKG, labs, and imaging Admit the patient to ICU for further observation	0:00

**Table t10-jetem-7-3-s1:** 

	Milestone	Did not achieve level 1	Level 1	Level 2	Level 3
1	**Emergency Stabilization (PC1)**	□ Did not achieve Level 1	□ Recognizes abnormal vital signs	□ Recognizes an unstable patient, requiring intervention Performs primary assessment Discerns data to formulate a diagnostic impression/plan	□ Manages and prioritizes critical actions in a critically ill patient Reassesses after implementing a stabilizing intervention
2	**Performance of focused history and physical (PC2)**	□ Did not achieve Level 1	Performs a reliable, comprehensive history and physical exam	□ Performs and communicates a focused history and physical exam based on chief complaint and urgent issues	□ Prioritizes essential components of history and physical exam given dynamic circumstances
3	**Diagnostic studies (PC3)**	□ Did not achieve Level 1	□ Determines the necessity of diagnostic studies	□ Orders appropriate diagnostic studies. Performs appropriate bedside diagnostic studies/procedures	□ Prioritizes essential testing Interprets results of diagnostic studies Reviews risks, benefits, contraindications, and alternatives to a diagnostic study or procedure
4	**Diagnosis (PC4)**	□ Did not achieve Level 1	□ Considers a list of potential diagnoses	□ Considers an appropriate list of potential diagnosis May or may not make correct diagnosis	□ Makes the appropriate diagnosis Considers other potential diagnoses, avoiding premature closure
5	**Pharmacotherapy (PC5)**	□ Did not achieve Level 1	□ Asks patient for drug allergies	□ Selects an medication for therapeutic intervention, consider potential adverse effects	□ Selects the most appropriate medication and understands mechanism of action, effect, and potential side effects Considers and recognizes drug-drug interactions
6	**Observation and reassessment (PC6)**	□ Did not achieve Level 1	□ Reevaluates patient at least one time during case	□ Reevaluates patient after most therapeutic interventions	□ Consistently evaluates the effectiveness of therapies at appropriate intervals
7	**Disposition (PC7)**	□ Did not achieve Level 1	□ Appropriately selects whether to admit or discharge the patient	□ Appropriately selects whether to admit or discharge Involves the expertise of some of the appropriate specialists	□ Educates the patient appropriately about their disposition Assigns patient to an appropriate level of care (ICU/Tele/Floor) Involves expertise of all appropriate specialists
9	**General Approach to Procedures (PC9)**	□ Did not achieve Level 1	□ Identifies pertinent anatomy and physiology for a procedure Uses appropriate Universal Precautions	□ Obtains informed consent Knows indications, contraindications, anatomic landmarks, equipment, anesthetic and procedural technique, and potential complications for common ED procedures	□ Determines a back-up strategy if initial attempts are unsuccessful Correctly interprets results of diagnostic procedure
20	**Professional Values (PROF1)**	□ Did not achieve Level 1	□ Demonstrates caring, honest behavior	□ Exhibits compassion, respect, sensitivity and responsiveness	□ Develops alternative care plans when patients’ personal beliefs and decisions preclude standard care
22	**Patient centered communication (ICS1)**	□ Did not achieve level 1	□ Establishes rapport and demonstrates empathy to patient (and family) Listens effectively	□ Elicits patient’s reason for seeking health care	□ Manages patient expectations in a manner that minimizes potential for stress, conflict, and misunderstanding. Effectively communicates with vulnerable populations, (at risk patients and families)
23	**Team management (ICS2)**	□ Did not achieve level 1	□ Recognizes other members of the patient care team during case (nurse, techs)	□ Communicates pertinent information to other healthcare colleagues	□ Communicates a clear, succinct, and appropriate handoff with specialists and other colleagues Communicates effectively with ancillary staff

## INSTRUCTOR MATERIALS

**Case 1 Title:** Cyanide Poisoning

**Case Description & Diagnosis (short synopsis):** A 31-year-old male, Jack Smith, is brought in by EMS from home after a house fire that occurred about 15 minutes prior to arrival. The patient’s story is that he lives by himself and that he was cooking something when he fell asleep in his bedroom. Upon awakening, he found that his house was filled with smoke and that he was trapped inside the house. Per EMS, the patient was found at the base of the stairs, laying on the ground. On arrival to the ED, the patient complains of headache, nausea, shortness of breath, palpitations, and dizziness. Vital signs note that the patient is tachycardic, tachypneic, and with an oxygen saturation of 98%. The physical examination reveals soot in the nares, singed facial hair, mild stridor, and obvious right ankle deformity.

**Equipment or Props Needed:**

- High-fidelity adult simulator
- Adult nasal cannula
- Adult non-rebreather mask (NRB)
- Adult Bag-Valve-Mask (BVM)
- IV supplies
- Intubation supplies - blades, endotracheal tube (ETT), syringe, bougie, colorimetric capnography
- Ventilator
- Splint Supplies
- Automated External Defibrillator (AED)

**Confederates needed:**

Nurse x2, Respiratory Therapy, Pharmacist, and EMS

**Stimulus Inventory:** [Table t2-jetem-7-3-s1]

**Background and brief information:** The case scenario takes place in a Level 1 Trauma Emergency Department. EMS calls in to report that they are en route to your facility with a 31-year-old male pulled from a house fire approximately 15 minutes ago. Per report, the patient was found at the base of the stairs. The patient is awake but confused, on 6L nasal cannula, and has an obvious deformity of the right ankle.

**Initial presentation:** The patient arrives at the emergency department complaining of headache, nausea, vomiting, shortness of breath, palpitation, and dizziness. Airway is intact, although stridor is noted. Patient has diffused wheezing in bilateral lung fields, and the patient is tachycardic. The patient appears to be confused and is therefore GCS documented as 14. There is soot in the nares, singed facial hair, mild stridor and an obvious right ankle deformity.

**How the scene unfolds:** The patient presents to the emergency room via EMS complaining of headache, nausea, vomiting, shortness of breath, heart palpitations, and dizziness. Upon arrival, the participants should place the patient on a monitor to obtain a full set of vital signs and start their trauma assessment of airway, breathing, circulation, disability, and exposure. Airway will be intact, although mild stridor is noted. Breath sounds will note diffused bilateral wheezing. Patient is currently on a 6L nasal cannula with a respiratory rate of 24 and an SpO2 of 98%. The patient’s heart rate is 132 beats per minute with 2+ pulses in all four extremities. The obvious deformity of the right ankle will be identified, but the patient denies pain in other extremities. The face exam will note soot in the nares and singed facial hair. A GCS of 14 will be calculated.

Simultaneously with the history and physical, the participants should request nursing staff to start IVs, begin administering fluids, and collect blood for testing. The respiratory therapist is concerned that the patient’s breathing is poor as they note tachypnea and stridor and request intervention. Participants should revisit the patient’s airway and breathing status. At this time, participants should upgrade the patient’s oxygen support to a non-rebreather or intubation. If the non-rebreather is chosen, the patient will become more lethargic, less responsive, and the stridor will get worse. If the patient is not intubated, the patient will go into severe respiratory failure and cardiac arrest.

While blood tests are being run, the respiratory therapist will obtain the arterial blood gas which notes a pH 7.1, CO2 20, HCO3 14. These results note metabolic acidosis and should trigger the participants to create a differential diagnosis including lactic acidosis from cyanide poisoning. Participants should then realize that the patient is not improving with the supplemental oxygen and recognize the need for a cyanide antidote. If the antidote is not given, the patient will go into asystole cardiac arrest.

Once the patient has a definitive airway and the cyanide antidote has been given, the participants should begin their secondary assessment including FAST exam, EKG, and imaging. Participants should also stabilize the ankle with splint material. Ultimately, the patient will need a consult to orthopedics and admission to the intensive care unit.

**Critical actions:**

1. Obtain full history from the patient/EMS
2. Obtain Vital Signs, recognize abnormalities and address
  - supplemental oxygen
  - fluids for tachycardia
3. Identify respiratory distress and increase oxygen support
  - Intubation will be required
4. Address the possibility of cyanide poisoning
5. Consult Poison Control Center and treat with antidote
6. Secondary Assessment - Order proper EKG, labs, and imaging
7. Admit the patient to ICU for further observation

**Case Title:** Cyanide Poisoning

**Chief Complaint:** headache, nausea, vomiting, shortness of breath, palpitation, and dizziness[Table t3-jetem-7-3-s1]

**General Appearance:** Moderate distress, confused

**Primary Survey:**

- **Airway:** intact - patient able to state his name but stridor present at rest
- **Breathing:** bilateral wheezing on auscultation
- **Circulation:** 2+ pulses x4 extremities; tachycardia

**History:**

- **History of present illness:** EMS calls in to report that they are en route to your facility with a 31-year-old male pulled from a house fire approximately 15 minutes ago. Per report, the patient was found at the base of the stairs. The patient is awake but confused, on 6L nasal cannula, and has an obvious deformity of the right ankle.
- **Past medical history**: None
- **Past surgical history:** None
- **Medications:** None
- **Allergies:** No known drug allergies
- **Social history:** Denies all

**Secondary Survey/Physical Examination:**

- **General appearance:** Moderate distress
- **HEENT:**
  ○ **Head:** Within normal limits, singed facial hairs
  ○ **Eyes:** Within normal limits
  ○ **Ears:** Within normal limits
  ○ **Nose:** Soot present
  ○ **Throat:** Soot present
- **Neck:** C collar placed per EMS
- **Heart:** Sinus tachycardia
- **Lungs:** Bilateral, diffused wheezing, on 6L nasal cannula
- **Abdominal/GI:** Within normal limits
- **Genitourinary:** Within normal limits
- **Rectal:** Within normal limits
- **Extremities:** 2+ pulses x4 extremities, R ankle deformity with significant pain to palpation, able to flex at right knee and hip without difficulty
- **Back:** Within normal limits
- **Neuro:** GCS - 14 (E4, V4, M6)
- **Skin:** Erythematous
- **Lymph:** Within normal limits
- **Psych:** Within normal limits[Table t4-jetem-7-3-s1]

[Table t5-jetem-7-3-s1] [Table t6-jetem-7-3-s1] [Table t7-jetem-7-3-s1] *Sinus Tachycardia ECG*

(example 1). Learn the Heart. https://www.healio.com/cardiology/learn-the-heart/ecg-review/ecg-archive/sinus-tachycardia-ecg-1. Published March 4, 2003. Accessed September 27, 2021. CC BY 4.0.

[Fig f1-jetem-7-3-s1] *Initial Chest X-Ray*

Stillwaterising. In Wikimedia Commons. https://commons.wikimedia.org/wiki/File:Chest_Xray_PA_3-8-2010.png. Published March 8, 2010. Accessed September 29, 2021. CC BY 1.0.

[Fig f2-jetem-7-3-s1] *Post-intubation Chest X-Ray*

In: EM Sim Cases. https://emsimcases.com/2015/11/24/dka/normal-post-intubation-cxr/. Accessed September, 29, 2021. CC BY-SA 4.0.[Fig f3-jetem-7-3-s1]

## OPERATOR MATERIALS

[Table t8-jetem-7-3-s1] **Diagnosis:**

Cyanide Poisoning

**Disposition:**

After intubation and treatment, the patient needs to be admitted to the ICU for further observation.

## DEBRIEFING AND EVALUATION PEARLS

### Cyanide Poisoning

**Background:**

When cyanide enters the body via inhalation, it blocks the cells from utilizing oxygen by binding to the cytochrome oxidase in the mitochondria.^2^ The inability of the cell to use oxygen forces cells from aerobic metabolism into anaerobic metabolism. Anaerobic metabolism results in the production of lactic acid, which causes metabolic acidosis.^3^ The human body cannot sustain with the lack of oxygen and anaerobic metabolism for a prolonged period of time. Ultimately, the body will suffer cardiorespiratory arrest.^1^

**Clinical Presentation:**

- Generalized weakness, nausea, confusion, headache, difficulty breathing, vertigo, and red flushing of the skin.
- Over time and in more severe cases, patients may experience seizures, cardiac arrest, and loss of consciousness.

**Workup finding:**

- Normal oxygen saturation levels
- Significantly elevated lactic acid levels

**Treatment:**

- Hydroxocobalamin.
  ○ IV infusion over the span of 15 minutes. A second 5g dosage may be administered with a varying time span of 15 minutes to 2 hours depending on clinical observation.^3^
- Sodium Thiosulfate.
  ○ Used to treat cyanide poisoning. While sodium thiosulfate is oftentimes effective, hydroxocobalamin has been found to be more effective especially in more severe cases.

**Pearls:**

The goal of this simulation is to inform emergency room providers about the dangers of smoke toxicity in fire victims. Through this case simulation, participants will be able to identify the symptoms of cyanide poisoning and be aware of the antidotes and the importance of beginning treatment prior to receiving lab results. Participants should also become more familiar with diagnosing and treating other common smoke toxicities including carbon monoxide. Learners should also be able to identify burns and injuries and know how to prioritize treatment.

**Other debriefing points:**

Many fire departments and emergency services have recently mandated that at least some of the EMS personnel carry the CYANOKIT (hydroxocobalamin). Their protocol is to administer the medication prior to arriving at the emergency department. Emergency department personnel should inquire if a Cyanokit has been administered.^6^

Research suggests that providers should begin treating suspected smoke inhalation victims with hydroxocobalamin immediately. If symptoms do not improve, or if cyanide poisoning is continued to be suspected, sodium thiosulfate can be administered in conjunction with hydroxocobalamin.^2^

## SIMULATION ASSESSMENT

*Cyanide Poisoning*

Learner: _________________________________________

***Assessment Timeline***

This timeline is to help observers assess their learners. It allows observer to make notes on when learners performed various tasks, which can help guide debriefing discussion.[Table t9-jetem-7-3-s1]

**Critical Actions:**

□ Obtain full history from the patient/EMS
□ Obtain Vital Signs, recognize abnormalities and address
  □ Supplemental oxygen
  □ F;uids for tachycardia
□ Identify respiratory distress and increase oxygen support
  □ Intubation will be required
□ Address the possibility of cyanide poisoning
□ Consult Poison Control Center and treat with antidote
□ Secondary Assessment - Order proper EKG, labs, and imaging
□ Admit the patient to ICU for further observation

**Summative and formative comments:**

**Milestones assessment:** [Table t10-jetem-7-3-s1]
