# Supplementary material for: Cyanide Poisoning
Source: J Educ Teach Emerg Med. 2022 Jul 15;7(3):S1–S25. doi: 10.21980/J80W76 (PMC10332703; doi:10.21980/J80W76)
Supplement: Supplementary file 1 [file jetem-7-3-s1-supp1.pptx]

## Slide 1
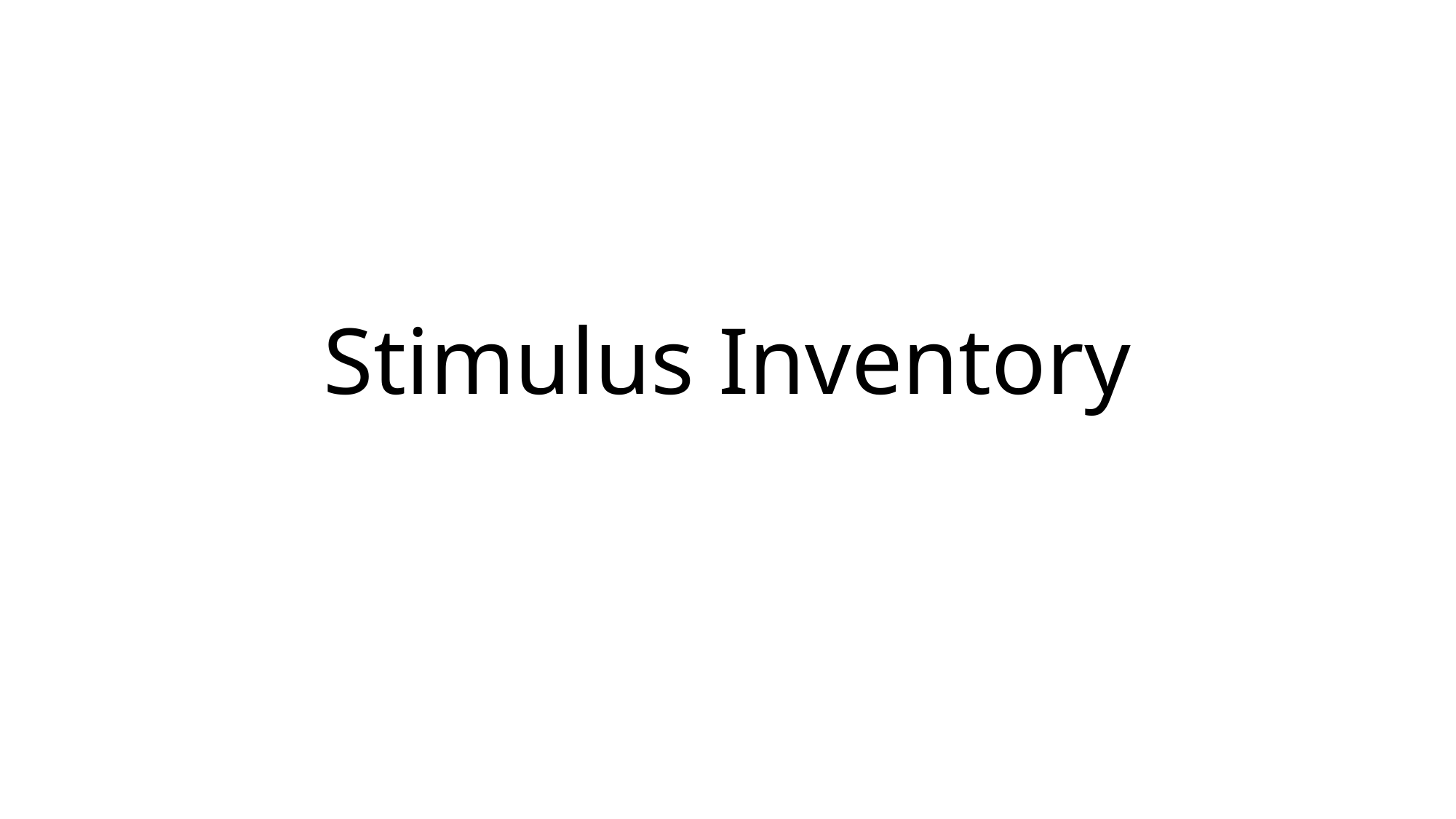

# Stimulus Inventory

## Slide 2
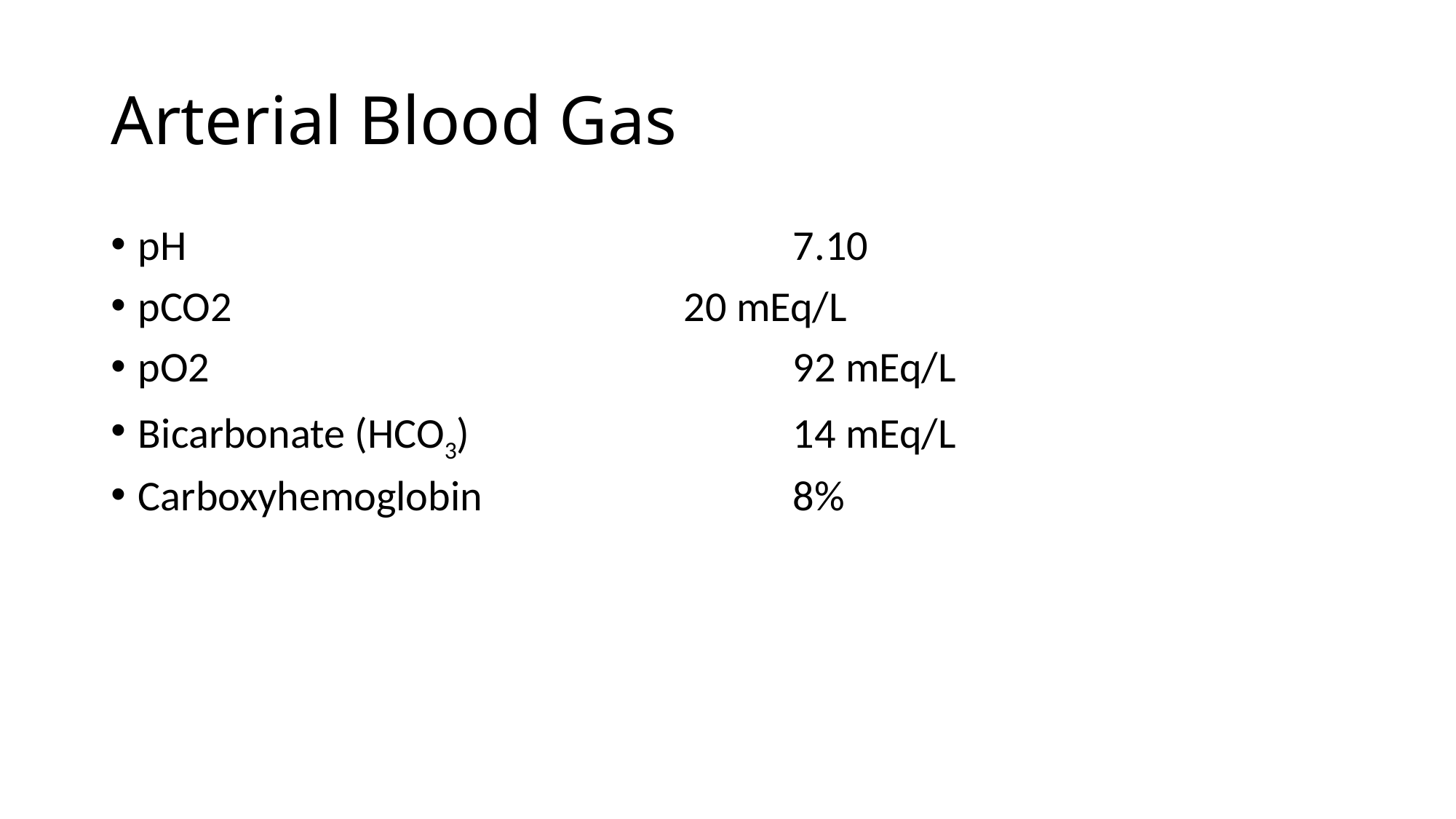

# Arterial Blood Gas
pH						7.10
pCO2					20 mEq/L
pO2						92 mEq/L
Bicarbonate (HCO3)			14 mEq/L
Carboxyhemoglobin			8%

## Slide 3
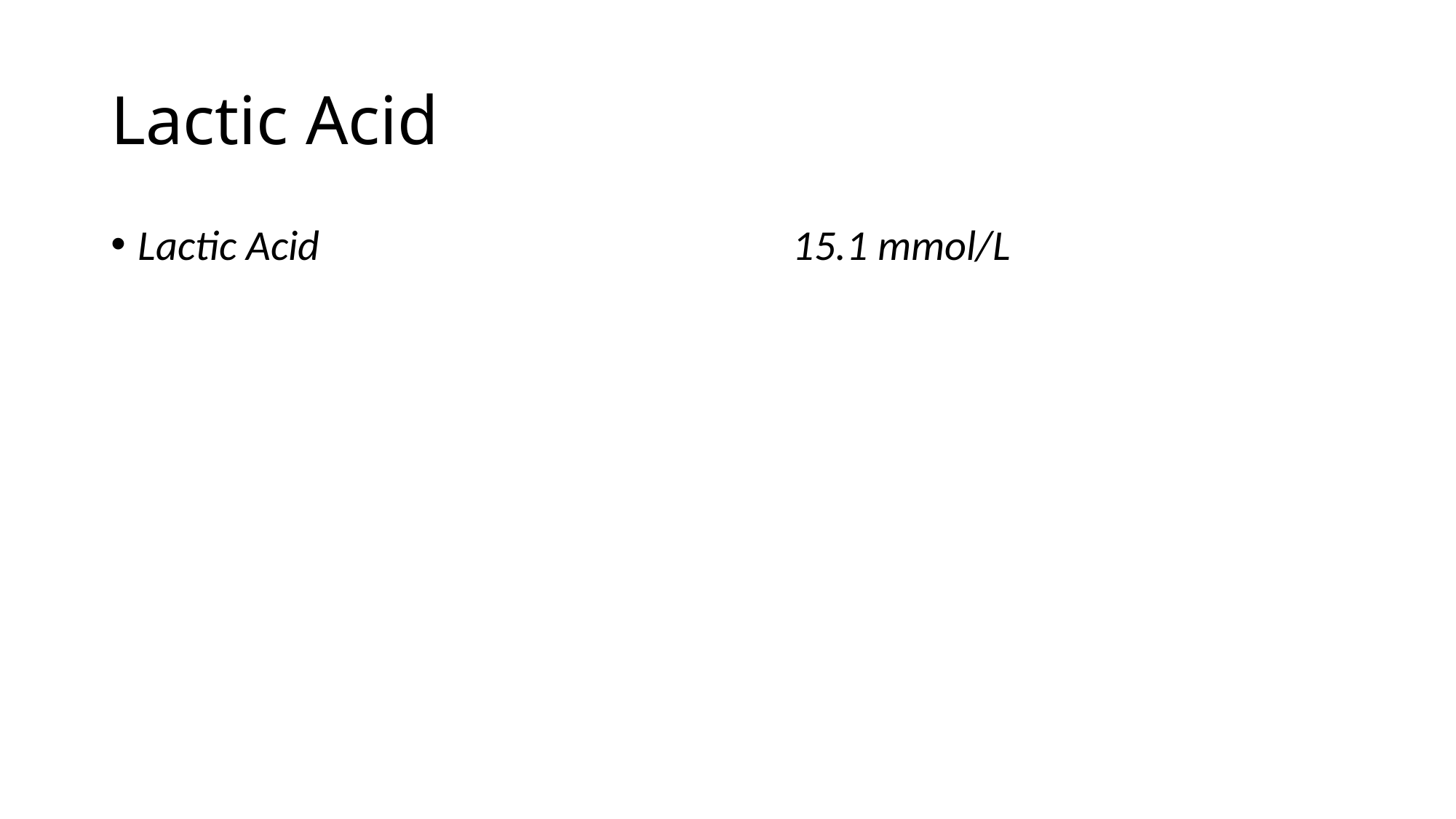

# Lactic Acid
Lactic Acid					15.1 mmol/L

## Slide 4
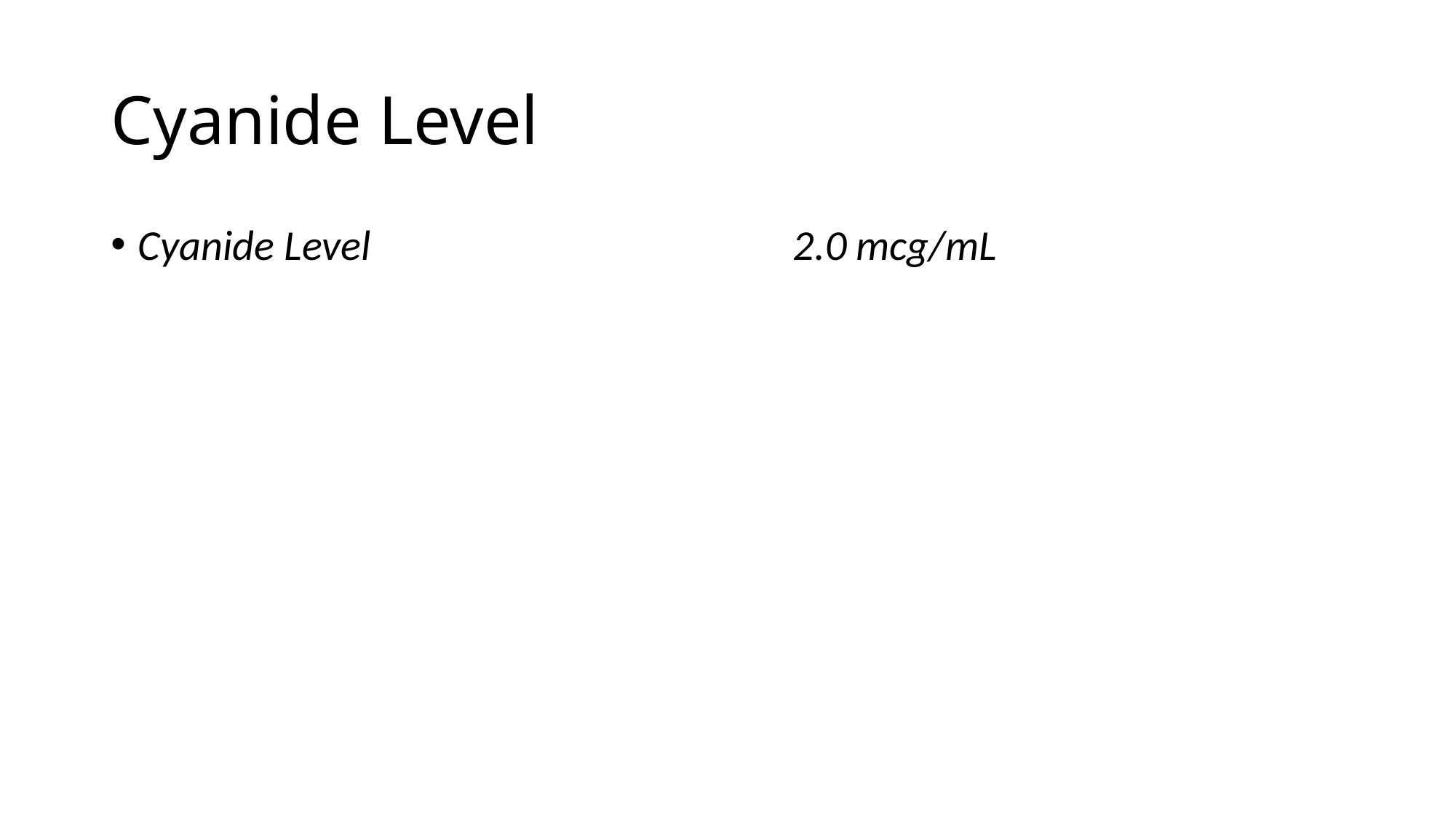

# Cyanide Level
Cyanide Level				2.0 mcg/mL

## Slide 5
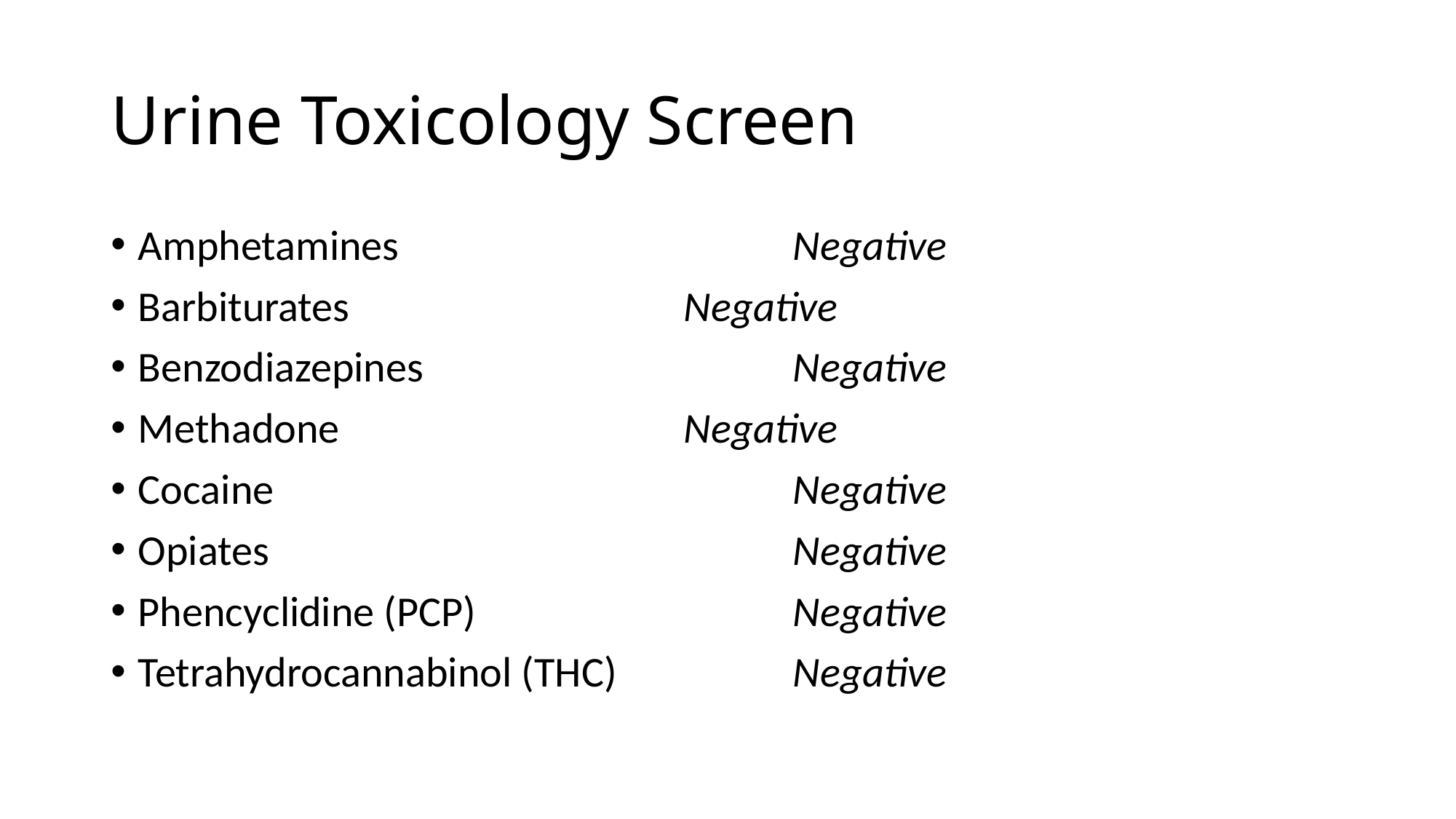

# Urine Toxicology Screen
Amphetamines				Negative
Barbiturates				Negative
Benzodiazepines				Negative
Methadone				Negative
Cocaine					Negative
Opiates					Negative
Phencyclidine (PCP)			Negative
Tetrahydrocannabinol (THC)		Negative

## Slide 6
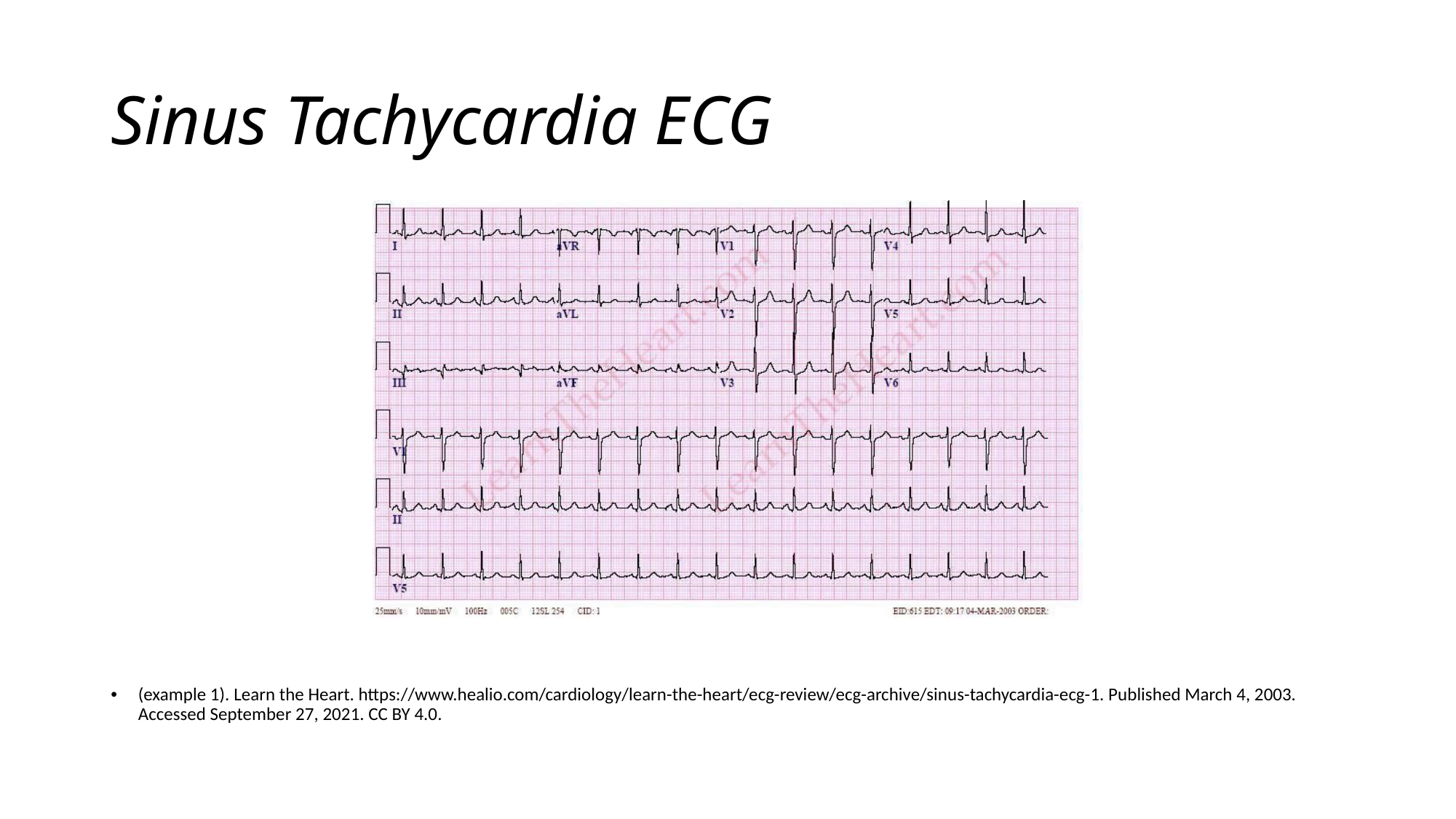

# Sinus Tachycardia ECG
(example 1). Learn the Heart. https://www.healio.com/cardiology/learn-the-heart/ecg-review/ecg-archive/sinus-tachycardia-ecg-1. Published March 4, 2003. Accessed September 27, 2021. CC BY 4.0.

## Slide 7
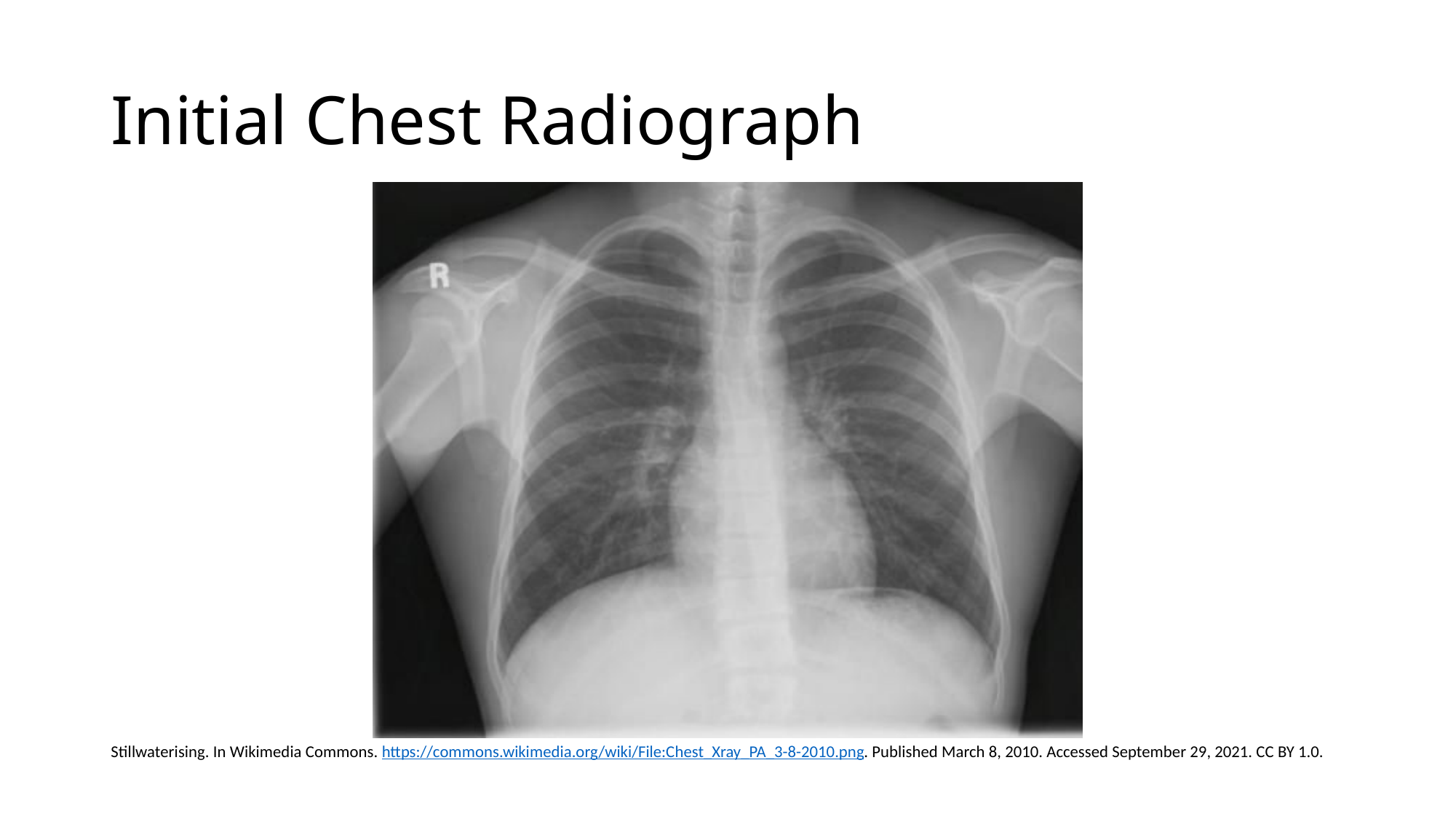

# Initial Chest Radiograph
Stillwaterising. In Wikimedia Commons. https://commons.wikimedia.org/wiki/File:Chest_Xray_PA_3-8-2010.png. Published March 8, 2010. Accessed September 29, 2021. CC BY 1.0.

## Slide 8
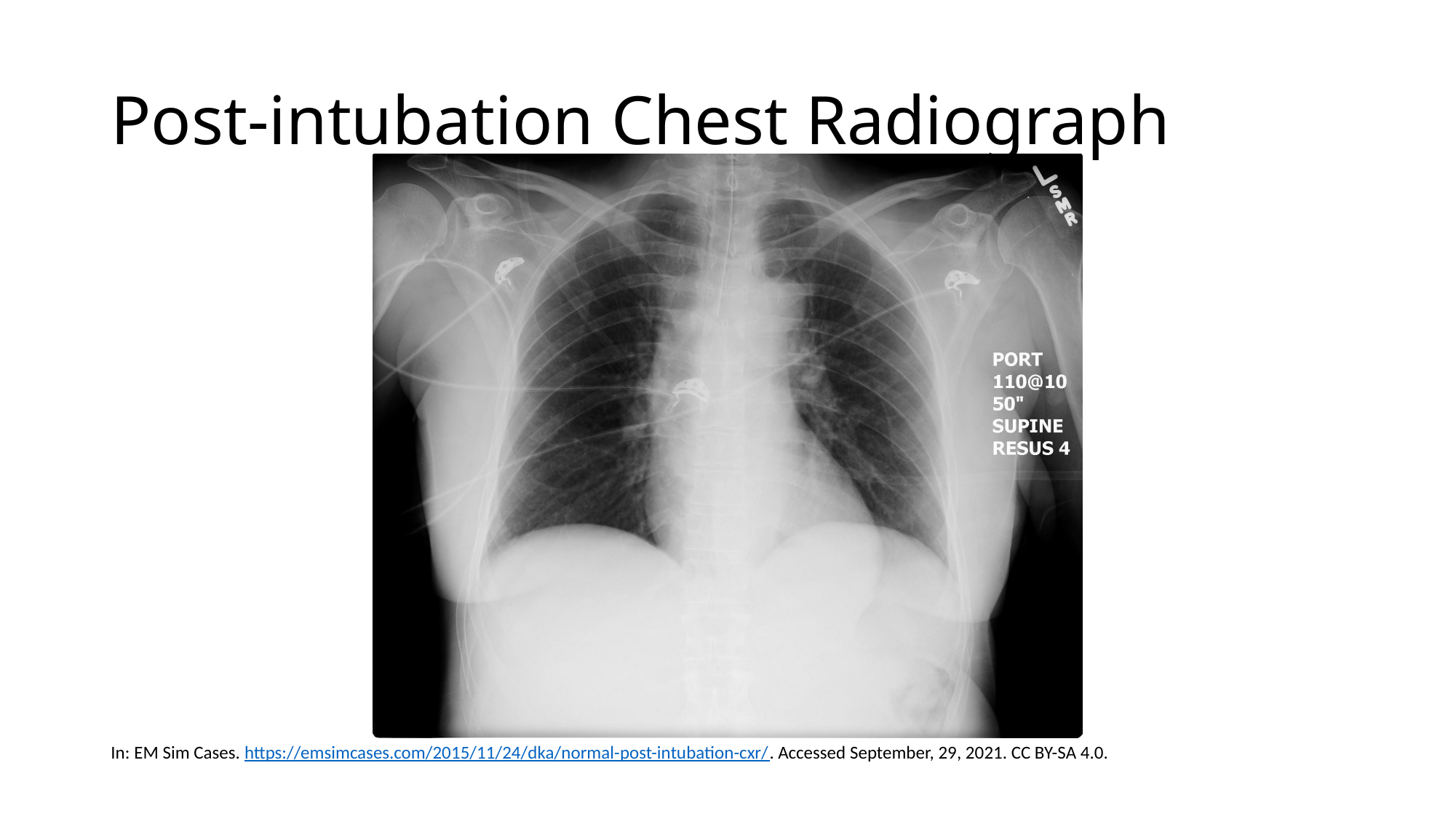

# Post-intubation Chest Radiograph
In: EM Sim Cases. https://emsimcases.com/2015/11/24/dka/normal-post-intubation-cxr/. Accessed September, 29, 2021. CC BY-SA 4.0.

## Slide 9
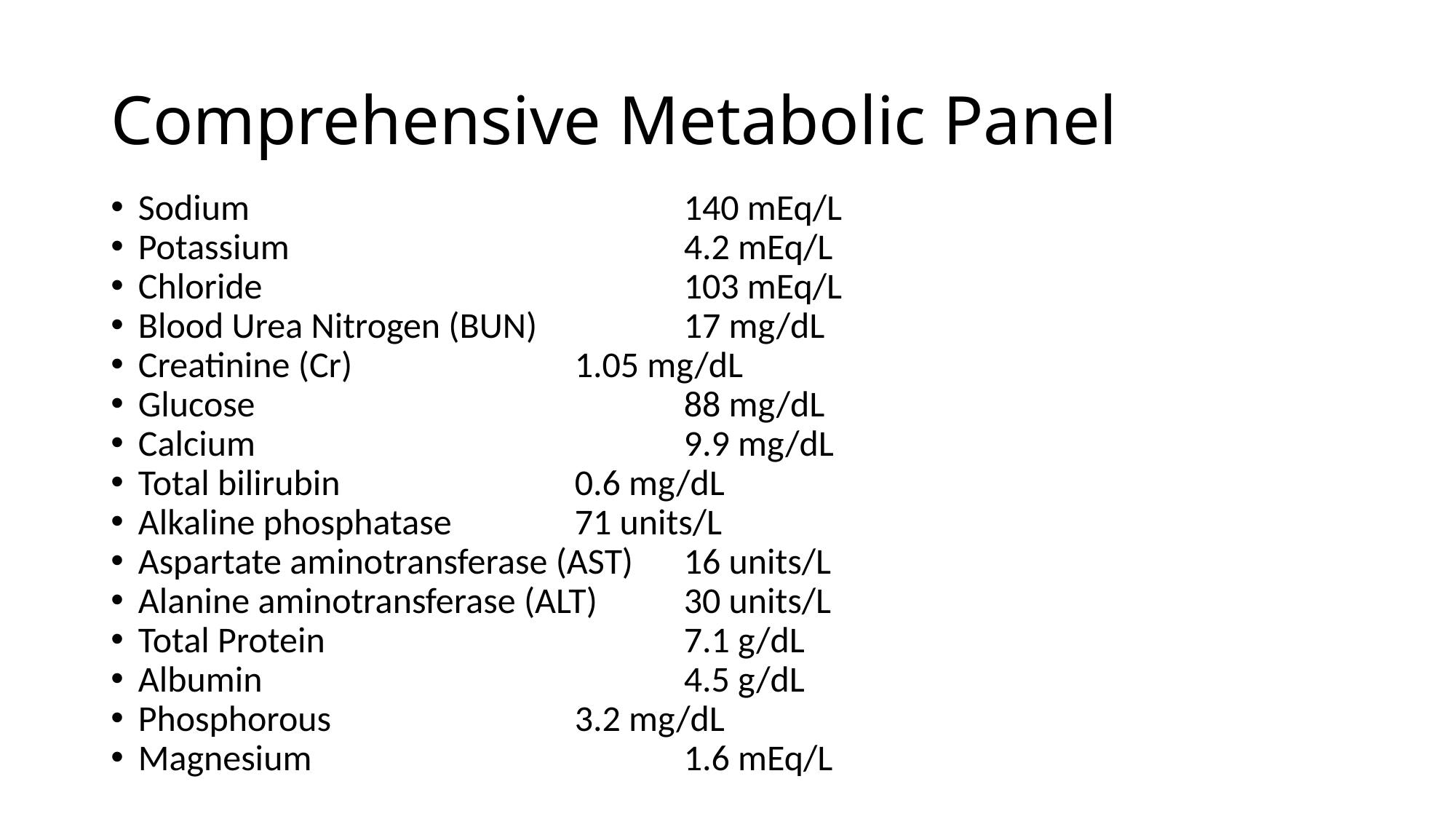

# Comprehensive Metabolic Panel
Sodium				140 mEq/L
Potassium				4.2 mEq/L
Chloride				103 mEq/L
Blood Urea Nitrogen (BUN)		17 mg/dL
Creatinine (Cr)			1.05 mg/dL
Glucose				88 mg/dL
Calcium				9.9 mg/dL
Total bilirubin			0.6 mg/dL
Alkaline phosphatase		71 units/L
Aspartate aminotransferase (AST)	16 units/L
Alanine aminotransferase (ALT)	30 units/L
Total Protein				7.1 g/dL
Albumin				4.5 g/dL
Phosphorous			3.2 mg/dL
Magnesium				1.6 mEq/L

## Slide 10
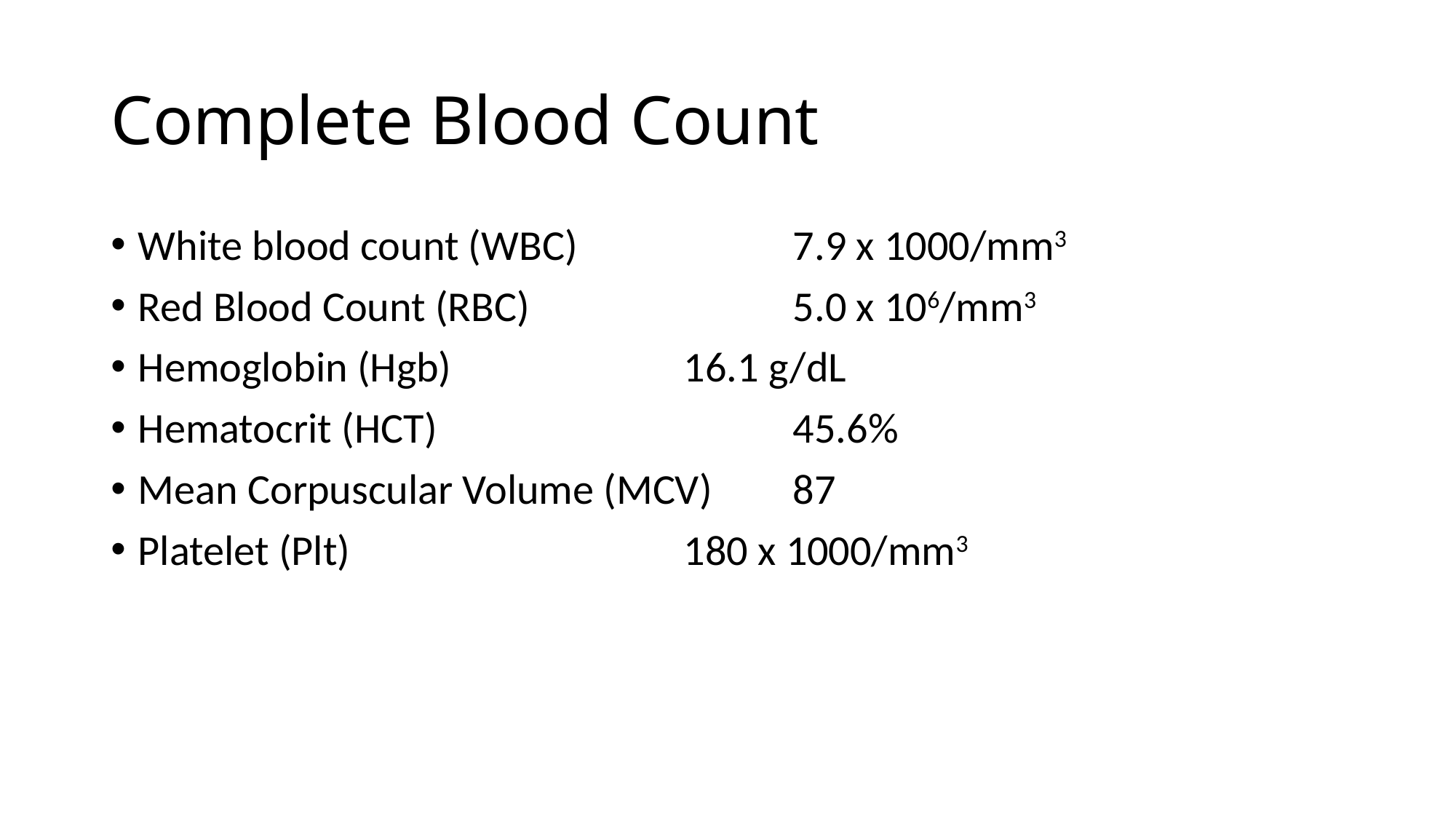

# Complete Blood Count
White blood count (WBC)		7.9 x 1000/mm3
Red Blood Count (RBC)			5.0 x 10^6^/mm3
Hemoglobin (Hgb)			16.1 g/dL
Hematocrit (HCT)				45.6%
Mean Corpuscular Volume (MCV)	87
Platelet (Plt)				180 x 1000/mm3
