# Supplementary figures and images for: Cyanide Poisoning
Source: J Educ Teach Emerg Med. 2022 Jul 15;7(3):S1–S25. doi: 10.21980/J80W76 (PMC10332703; doi:10.21980/J80W76)

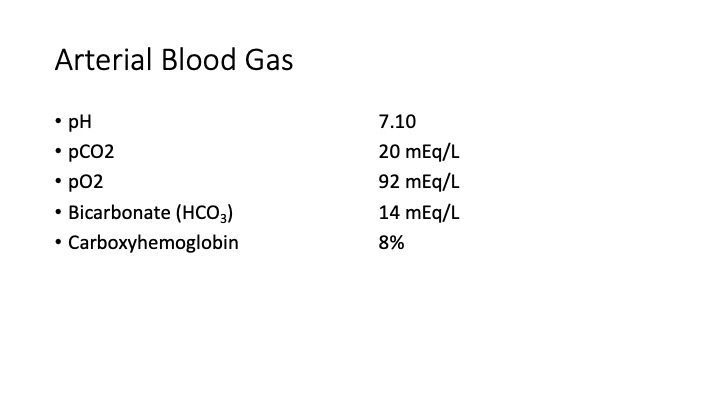

Supplement: Supplementary file 2 [file jetem-7-3-s1-supp2.jpeg]

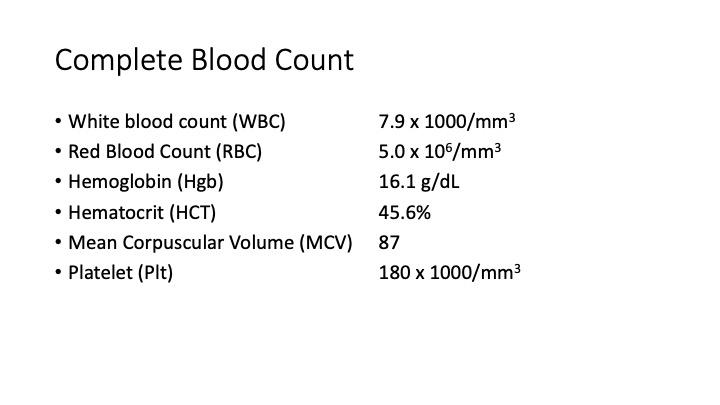

Supplement: Supplementary file 3 [file jetem-7-3-s1-supp3.jpeg]

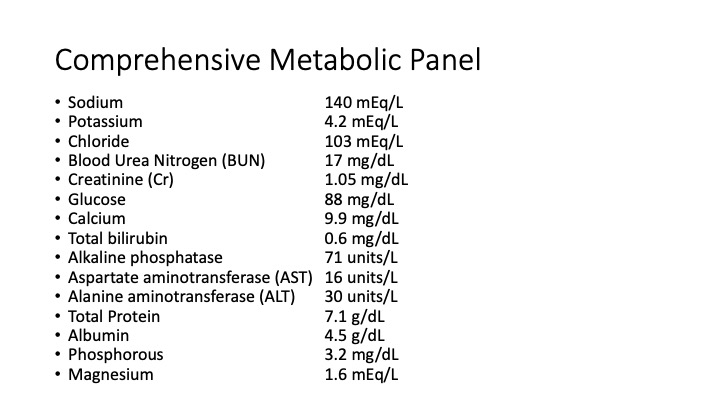

Supplement: Supplementary file 4 [file jetem-7-3-s1-supp4.jpeg]

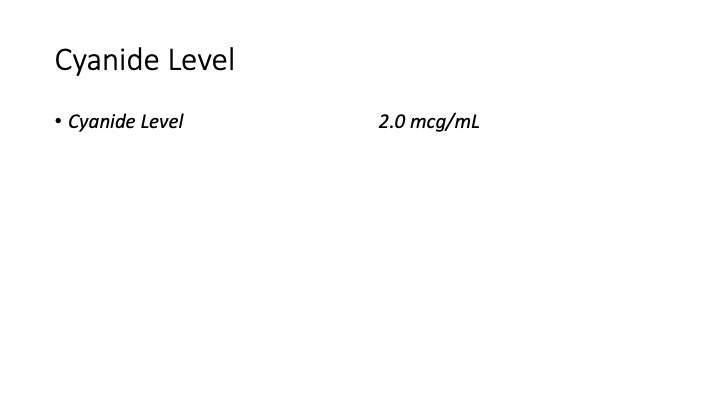

Supplement: Supplementary file 5 [file jetem-7-3-s1-supp5.jpeg]

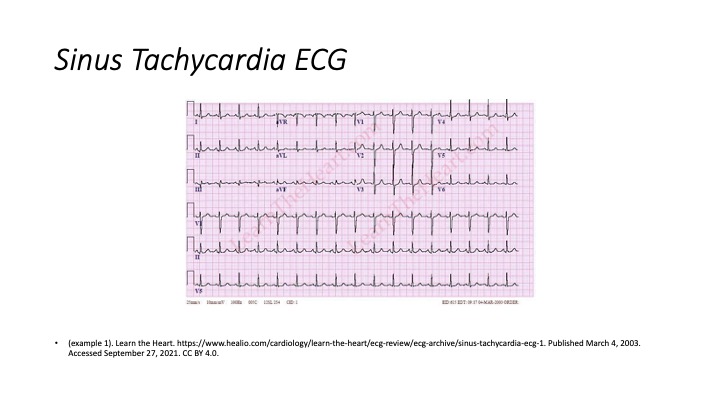

Supplement: Supplementary file 6 [file jetem-7-3-s1-supp6.jpeg]

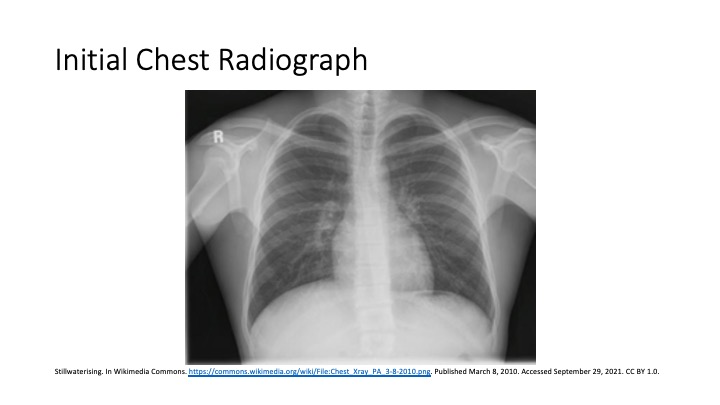

Supplement: Supplementary file 7 [file jetem-7-3-s1-supp7.jpeg]

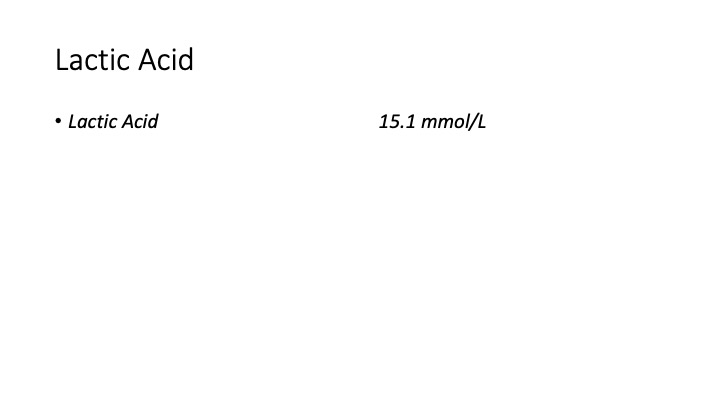

Supplement: Supplementary file 8 [file jetem-7-3-s1-supp8.jpeg]

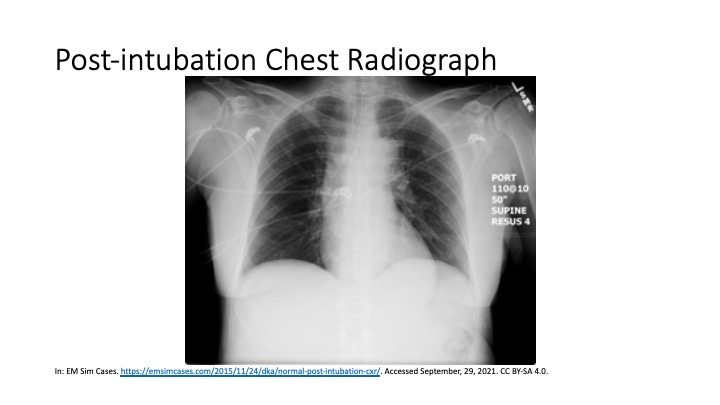

Supplement: Supplementary file 9 [file jetem-7-3-s1-supp9.jpeg]

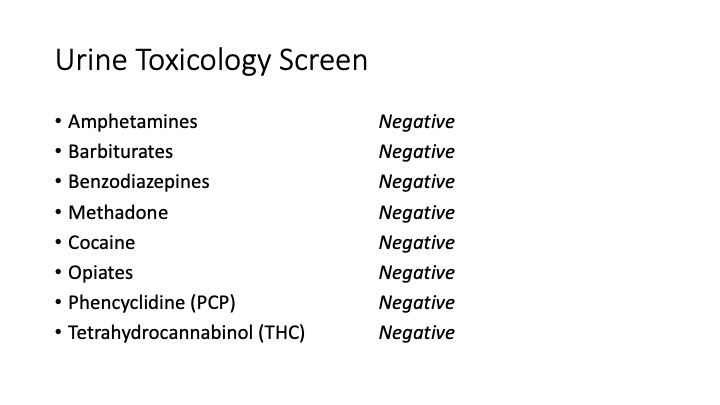

Supplement: Supplementary file 10 [file jetem-7-3-s1-supp10.jpeg]
